# Impact of insurance and neighborhood socioeconomic status on clinical outcomes in therapeutic clinical trials for breast cancer

**DOI:** 10.1002/cam4.3542

**Published:** 2020-12-02

**Authors:** Samilia Obeng‐Gyasi, Anne O’Neill, Fengmin Zhao, Sheetal M. Kircher, Timisina R. Lava, Lynne I. Wagner, Kathy D. Miller, Joseph DA. Sparano, George W. Sledge, Ruth C. Carlos

**Affiliations:** ^1^ Division of Surgical Oncology The Ohio State University Wexner Medical Center and James Comprehensive Cancer Center Columbus OH USA; ^2^ Dana Farber Cancer Institute–ECOG‐ACRIN Biostatistics Center Boston MA USA; ^3^ Northwestern University Robert H. Lurie Comprehensive Cancer Center Chicago IL USA; ^4^ Department of Surgery Indiana University School of Medicine Indianapolis IN USA; ^5^ Department of Social Sciences & Health Policy Wake Forest School of Medicine; Wake Forest Baptist Comprehensive Cancer Center Winston‐Salem NC USA; ^6^ Indiana University School of Medicine|Melvin and Bren Simon Cancer Center Indianapolis IN USA; ^7^ Montefiore Medical Center Albert Einstein College of Medicine Albert Einstein Cancer Center Bronx NY USA; ^8^ Stanford University Palo Alto CA USA; ^9^ Comprehensive Cancer Center University of Michigan Ann Arbor MI USA

**Keywords:** breast cancer, clinical trials, insurance

## Abstract

The objective of this study was to evaluate the impact of insurance and neighborhood SES (nSES) on chemotherapy completion and overall mortality among participants in breast cancer clinical trials. The data sources for this study were two adjuvant breast cancer trials (ECOG E1199 and E5103) collectively including 9790 women. Insurance status at trial registration was categorized into private, government (Medicaid, Medicare, and other government type insurance), and self‐pay. An Agency for Healthcare Research Quality (AHRQ) nSES index was calculated using residential zip codes linked to county level data on occupation, income, poverty, wealth, education, and crowding. Logistic regression and Cox Proportional Hazard models estimated odds ratios (OR) for chemotherapy treatment completion and hazard ratios (HR) for mortality, respectively, for insurance status and nSES. The models adjusted for: race, age, tumor size, nodal status, hormone receptor status, and primary surgery. The majority of patients had private insurance at trial registration: E1199: 85.6% (4154/4854) and E5103: 82.4% (3987/4836); median SES index was 53.8 (range: 41.8‐66.8) and 54.1 (range: 44.5‐66.1), respectively. Patients with government insurance were less likely to complete chemotherapy treatment (E1199 OR (95%CI): 0.73 (0.57‐0.94); E5103 0.76 (0.64‐0.91)) and had an increased risk of death (E1199 HR (95%CI): 1.44 (1.22‐1.70); E5103 1.29 (1.06‐1.58)) compared to the privately insured patients. There was no association between nSES and chemotherapy completion or overall mortality. Patients with government insurance at trial registration appeared to face barriers in chemotherapy completion and had a higher overall mortality compared to their privately insured counterparts.

## 1 INTRODUCTION

Social determinants of health (SDH) such as education, neighborhood and housing, transportation, economic stability, food, and healthcare systems have been shown to powerfully influence clinical outcomes.^1^ Particularly, area of residence and insurance consistently impact stage of presentation, treatment, and mortality in breast cancer patients.^2^, ^3^, ^4^, ^5^, ^6^ To date, the majority of work evaluating SDH have been in nonclinical trial populations (i.e. patients not actively enrolled in a clinical trial). Research on SDH and clinical trials have focused on patient enrollment, demonstrating that participants are more likely to be younger, white, have high SES and are privately insured.^7^, ^8^ There are few studies evaluating the effect of SDH on continued trial participation, treatment completion, and clinical outcomes such as survival.^9^ This knowledge gap is significant as clinical trials provide a population of patients with similar tumor biology access to healthcare and equivalent treatment modalities. Hypothetically, clinical trials should provide an environment that can be leveraged to mitigate the effects of SDH, reduce disparities in clinical outcomes and create health and healthcare equity.

The objective of this study was to understand the relationship between insurance status and neighborhood socioeconomic status (nSES) at the time of trial registration and the clinical endpoints of completion of trial chemotherapy and overall mortality among breast cancer patients enrolled in clinical trials. Insurance status has specific eligibility criteria, that is, income or employment, and more closely reflects a patient's individual SES; nSES reflects the built environment and its resource (e.g. transportation, access to healthcare, and food options). Additionally, nSES may act independently of individual SES. Therefore, both area level SDH (nSES) and individual level SDH (insurance type) are used to evaluate SES. We hypothesize that due to similarities in tumor biology coupled with highly regulated treatment algorithms in clinical trials, insuranceand nSES will not affect either clinical outcome.

## 2 METHODS

### 2.1 Study database

This study is a retrospective review of prospectively collected data from two large randomized adjuvant breast cancer clinical trials conducted by ECOG‐ACRIN: ECOG E1199 and ECOG E5103. ECOG E1199 compared the efficacy of administering four cycles of docetaxel or paclitaxel either weekly or every 3 weeks after four cycles of doxorubicin and cyclophosphamide (AC) among women with stage II–III breast cancer (see supplementary materials for protocol).^10^, ^11^ ECOG E5103 compared doxorubicin and cyclophosphamide (AC) for four cycles, followed by 12 weeks of weekly paclitaxel with placebo (Arm A) to the same chemotherapy with either concurrent bevacizumab (Arm B) or with concurrent plus sequential bevacizumab (Arm C) among women with node positive or high‐risk node negative HER2 negative disease (see supplementary materials for protocol).^12^ E1199 accrued patients from October 1999 to January 2002 and E5103 from November 2007 to February 2011.

### 2.2 Insurance status

Insurance status at time of trial registration for this population consisted of private, Medicare +private, Medicaid, Medicare, Medicaid +Medicare, military, VA, National Health Service, no means to pay, and self‐pay.

### 2.3 SES index

An index of neighborhood level SES was created by linking the patient's home zip code at registration to county level data using 2016‐2017 Health Resource and Services Administration (HRSA) Area Health Resources File. The SES index, developed by Agency for Healthcare Research and Quality (AHRQ), is a weighted composite variable that includes occupation, income, poverty, wealth, education, and crowding.^13^, ^14^ When a zip code represented multiple counties, for each component variables in SES index, aggregate means and totals from those multiple counties were used to represent the county level estimates for that zip code.^15^

### 2.4 Statistical analysis

Two outcomes were of interest: (a) Completion of trial chemotherapy and (b) Survival (OS). Patients in E1199 coded as “yes” completed chemotherapy if they received taxane for four cycles, regardless of dose reduction and delay. Since more patients assigned to the bevacizumab containing arms in E5103 discontinued treatment early, this outcome for E5103 was coded as “yes” if patients completed the specified 4 cycles of AC and 12 cycles of paclitaxel (since this was similar across arms). Survival was defined as time from trial registration to date of death, otherwise patients were censored at date last known alive.

Chi‐square (for categorical) and Wilcoxon rank‐sum tests (for continuous variables) were used to assess the relationship between baseline demographic and disease characteristic variables and chemotherapy completion. Univariate and multivariate logistic regression and Cox Proportional Hazard models were used to estimate odds ratios (OR) for chemotherapy completion and hazard ratios (HR) for OS, respectively. Estimates for insurance status and nSES in the multivariate models were adjusted for: race, age, tumor size, nodal status, hormone receptor status (estrogen, progesterone), Human epidermal growth factor (HER 2) (in E1199), and primary surgery at baseline.

## 3 RESULTS

### 3.1 Study population

The total study population included n = 4954 patients from E1199 and n = 4836 patients from E5103. The median age (range) was 51 years (19‐84) and 51.7 years (21.2‐85.0) in E1199 and E5103, respectively. The majority of the study participants identified as white (E1199 84% (4183/4954), E5103 85% (4089/4836). Most patients had private insurance at trial registration: 85.6% (4154/4854) and 82.4% (3987/4836) and the median (range) SES index was 53.8 (41.8‐66.8) and 54.1 (44.5‐66.1), respectively (Table 1).

**TABLE 1:** Baseline demographics and disease characteristics by chemotherapy completion (n, col %).

Variable	E1199			E5103^b^		
	Completed chemotherapy			Completed chemotherapy^a^		
	No	Yes	Total	No N = 1868	Yes N = 2968	Total N = 4836
Treatment arm						
P3	110 (14.1)	1124 (27.4)	1234 (25.3)	—	—	—
P1	158 (20.2)	1053 (25.7)	1211 (24.8)	—	—	—
D3	196 (25.1)	1017 (24.8)	1213 (24.9)	—	—	—
D1	317 (40.6)	900 (22.0)	1217 (25.0)	—	—	—
A	—	—	—	274 (15)	698 (24)	972 (20)
B	—	—	—	773 (41)	1149 (39)	1922 (40)
C	—	—	—	821 (44)	1121 (38)	1942 (40)
Race						
White	652 (83.5)	3477 (84.9)	4129 (84.7)	1522 (82)	2567 (87)	4089 (85)
Black	77 (9.9)	327 (8.0)	404 (8.3)	267 (14)	277 (9)	544 (11)
Other	52 (6.6)	290 (7.1)	342 (7.0)	73 (4)	113 (4)	186 (4)
Age groups						
<40	81 (10.4)	495 (12.1)	576 (11.8)	216 (12)	393 (13)	609 (13)
40‐65	548 (70.2)	3183 (77.8)	3731 (76.5)	1413 (76)	2362 (80)	3775 (78)
>=65	152 (19.5)	416 (10.2)	568 (11.7)	239 (13)	213 (7)	452 (9)
Insurance type						
Private	611 (80.3)	3483 (86.7)	4094 (85.7)	1498 (81)	2489 (85)	3987 (84)
Government	135 (17.7)	459 (11.4)	594 (12.4)	307 (17)	339 (12)	646 (14)
Self‐pay	15 (2.0)	75 (1.9)	90 (1.9)	39 (2)	88 (3)	127 (3)
AHRQ SES Index Score (median, range)	53.8 (41.8, 66.8)	53.5 (44.1, 64.3)	53.7 (41.8, 66.8)	53.9 (45.2‐65.8)	54.3 (44.5‐66.1)	54.1 (44.5‐66.1)
Tumor size						
<=2 cm	278 (36.0)	1500 (37.0)	1778 (36.8)	746 (40)	1122 (38)	1868 (39)
>2 cm	495 (64.0)	2558 (63.0)	3053 (63.2)	1119 (60)	1845 (62)	2964 (61)
Nodal status						
Negative	120 (15.5)	443 (10.9)	563 (11.6)	536 (29)	782 (26)	1318 (27)
Positive	656 (84.5)	3633 (89.1)	4289 (88.4)	1331 (71)	2185 (74)	3516 (73)
HR status						
Negative	219 (28.0)	1168 (28.5)	1387 (28.4)	673 (36)	1071 (36)	1744 (36)
Positive	562 (72.0)	2926 (71.5)	3488 (71.6)	1193 (64)	1897 (64)	3090 (64)
Breast surgery						
BCS	317 (40.8)	1585 (38.9)	1902 (39.2)	864 (46)	1347 (45)	2211 (46)
Mastectomy	459 (59.2)	2492 (61.1)	2951 (60.8)	1004 (54)	1621 (55)	2625 (54)
HER2 status						
Negative	552 (80.7)	2853 (77.6)	3405 (78.1)	—	—	—
Positive	132 (19.3)	824 (22.4)	956 (21.9)	—	—	—

^a^ This outcome for E5103 was coded as “yes” if patients completed the 4 cycles of AC and the 12 cycles of paclitaxel (since this was similar across arms).

^b^ Any missing values for variables were excluded from calculations.

^c^ E1199 study arms: P1 weekly Paclitaxel, P3 Paclitaxel every 3 weeks, D1 weekly docetaxel D3 docetaxel every 3 weeks (see section 2.1 for full description).

^d^ E5103 study arms: Arm A 2 weeks of weekly paclitaxel with placebo, Arm B concurrent bevacizumab, Arm C concurrent plus sequential bevacizumab (see section 2.1 for full description)

Due to concerns of differential outcomes for patients with Medicaid versus Medicare, additional analyses (not shown) were conducted which showed no differential outcomes for patients with Medicaid versus Medicare, as well as no differential outcomes for patients with Medicaid or Medicare versus other types of government insurance. Given those results, insurance status at time of trial registration was categorized into three groups: private (including Medicare +private), government, and self‐pay. The government insurance group was a combination of patients with Medicaid, Medicare, Medicaid +Medicare, other government insurance (military, VA, National Health Service), and no means to pay.

### 3.2 Completion of trial chemotherapy

Overall, n = 4875 patients in E1199 started chemotherapy and 84% (4094) completed chemotherapy per protocol; 61.4% (2968/4836) of patients who started chemotherapy in E5013 completed it as specified. Patients with government type insurance at trial registration were less likely to complete chemotherapy relative to patients with private insurance (OR, .95 Confidence Interval (CI): E1199: 0.73 (0.57, 0.94); E5103: 0.76 (0.64‐0.91). There was no difference in chemotherapy completion between those who were self‐pay and the privately insured. There was also no association between nSES index with chemotherapy completion in either trial (Table 2).

**TABLE 2:** Logistic regression for chemotherapy completion^b^

	E1199		E5103^a^	
	OR (0.95 CI)		OR (0.95 CI)	
	Univariate	Multivariable	Univariate	Multivariable
Insurance type				
Government vs private	0.60 (0.48, 0.74)	0.73 (0.57, 0.94)	0.67 (0.56‐0.79)	0.76 (0.64‐0.91)
Self‐pay vs private	0.88 (0.50, 1.54)	1.00 (0.52, 1.94)	1.36 (0.93‐1.99)	0.98 (0.60‐1.61)
SES index (continuous)	1.04 (1.01, 1.06)	1.03 (1.01,1.06)	1.04 (1.02‐1.06)	1.03 (1.01‐1.05)
Race				
Black vs White	0.80 (0.61, 1.03)	0.92 (0.68, 1.25)	0.62 (0.51‐0.74)	0.61 (0.51‐0.74)
Other vs White	1.04 (0.77, 1.42)	1.12 (0.78, 1.60)	0.92 (0.68‐1.24)	0.83 (0.61‐1.13)
Age				
40‐65 vs <40	0.95 (0.74, 1.22)	0.95 (0.72, 1.25)	0.92 (0.77‐1.10)	0.89 (0.74‐1.07)
>=65 vs 40	0.45 (0.33, 0.60)	0.45 (0.32, 0.63)	0.49 (0.38‐0.63)	0.47 (0.36‐0.61)
Tumor size (>2 cm vs <=2 cm)	0.96 (0.82, 1.12)	1.01 (0.83, 1.23)	1.10 (0.97‐1.23)	1.12 (0.99‐1.27)
Nodal status (positive vs negative)	1.50 (1.21, 1.86)	1.79 (1.39, 2.32)	1.12 (0.99‐1.28)	1.20 (1.03‐1.40)
HR status (positive vs negative)	0.98 (0.82, 1.16)	0.92 (0.76, 1.13)	0.99 (0.89‐1.13)	0.89 (0.78‐1.04)
Primary surgery (mastectomy vs BCS)	1.08 (0.93, 1.27)	1.09 (0.91, 1.30)	1.03 (0.92‐1.16)	0.99 (0.87‐1.12)
Her2 status (positive vs negative)	1.21 (0.98, 1.48)	1.26 (1.01, 1.56)	—	—

^a^ This outcome for E5103 was coded as “yes” if patients completed the 4 cycles of AC and the 12 cycles of paclitaxel (since this was similar across arms).

^b^ Any missing values for variables were excluded from calculations.

### 3.3 Overall mortality

There was an association between patients with government type insurance at trial registration and an increased risk of mortality relative to patients with private insurance (OR, .95 CI: E1199: 1.44 (1.22, 1.70); E5103: 1.29 (1.06‐1.58)). There was no difference in overall mortality between self‐pay and privately insured patients. There was also no association for nSES index with OS in either trial (Table 3).

**TABLE 3:** Cox proportional hazard models for overall survival^a^

	E1199		E5103	
	HR (0.95 CI)		HR (0.95 CI)	
	Univariate	Multivariable	Univariate	Multivariable
Insurance type				
Government vs private	1.62 (1.40, 1.88)	1.44 (1.22, 1.70)	1.55 (1.28‐1.87)	1.29 (1.06‐1.58)
Self‐pay vs private	1.55 (1.08, 2.24)	1.40 (0.94, 2.10)	0.95 (0.56‐1.62)	1.17 (0.63‐2.20)
SES index (continuous)	0.98 (0.96, 1.00)	1.00 (0.98, 1.01)	0.97 (0.95‐0.99)	0.98 (0.96‐1.00)
Race				
Black vs White	1.38 (1.15, 1.66)	1.25 (1.02, 1.52)	1.27 (1.02‐1.58)	1.11 (0.88‐1.39)
Other vs White	0.88 (0.69, 1.11)	0.77 (0.58, 1.01)	0.87 (0.58‐1.31)	0.86 (0.56‐1.32)
Age				
40‐65 vs <40	0.88 (0.74, 1.05)	1.00 (0.83, 1.21)	0.89 (0.71‐1.11)	1.00 (0.79‐1.26)
>=65 vs 40	1.54 (1.25, 1.89)	1.59 (1.26, 2.01)	1.58 (1.19‐2.08)	1.68 (1.26‐2.25)
Tumor size (>2 cm vs <=2 cm)	1.75 (1.55, 1.98)	1.79 (1.55, 2.06)	1.89 (1.60‐2.23))	1.78 (1.50‐2.12)
Nodal status (positive vs negative)	1.92 (1.54, 2.39)	2.44 (1.91, 3.12)	1.71 (1.41‐2.06)	2.36 (1.91‐2.91)
HR status (positive vs negative)	0.66 (0.58, 0.74)	0.64 (0.56, 0.73)	0.72 (0.62‐0.83)	0.48 (0.40‐0.56)
Primary surgery (mastectomy vs BCS)	1.45 (1.29, 1.63)	1.24 (1.09, 1.42)	1.48 (1.27‐1.72)	1.31 (1.12‐1.53)
Her2 status (positive vs negative)	1.05 (0.91, 1.20)	1.00 (0.87, 1.16)	—	—
Treatment arm^b^			—	—
P1 vs P3	0.88 (0.76, 1.03)	0.89 (0.75, 1.05)	—	—
D3 vs P3	0.86 (0.74, 1.00)	0.85 (0.72, 1.00)	—	—
D1 vs P3	1.01 (0.87, 1.18)	0.94 (0.80, 1.12)	—	—

^a^ Any missing values for variables were excluded from calculations.

^b^ E5103: no differences by treatment arm with respect to mortality. ^c^E1199 study arms: P1 weekly Paclitaxel, P3 Paclitaxel every 3 weeks, D1 weekly docetaxel D3 docetaxel every 3 weeks (see section 2.1 for full description)

## 4 DISCUSSION

Insurance type at trial registration is associated with clinical outcomes among breast cancer patients enrolled in E1199 and E5103. Specifically, participants with government insurance at trial registration were less likely to complete the clinical trial chemotherapy regimen and had a higher risk of overall mortality than those with private insurance. There was no association between patients with self‐pay insurance or nSES and clinical trial chemotherapy completion or with overall mortality.

Multiple studies have confirmed the relationship between insurance and clinical outcomes among breast cancer patients in nonclinical trial settings. Privately insured breast cancer patients are more likely to present with an earlier disease stage, receive guideline concordant care and have an increased overall survival compared to their uninsured or government insured counterparts.^3^, ^16^, ^17^, ^18^, ^19^ Moreover, differences in insurance status have been found to contribute one‐third of the excess risk of death among nonelderly black breast cancer patients compared to their white counterparts.^18^ Of note, in the aforementioned study, black women had higher rates of government insurance (or no insurance) than white women. A possible explanation for worse outcomes among Medicaid and uninsured patients include advanced disease stages at presentation and higher comorbidities due to poor healthcare access.^20^ Furthermore, due to low financial reserves, Medicaid and uninsured patients face higher levels of financial hardship which can result in adverse behaviors (i.e. treatment nonadherence) that offset treatment cost but worsen survival.^4^, ^21^, ^22^

Scant literature exists on the relationship of insurance and outcomes in clinical trial settings. In a recent study by Unger et al., in clinical trials that lengthened survival, Medicaid and uninsured patients derived no survival benefit compared to the privately insured.^9^ Additionally, the association between insurance, progression, or relapse free survival and overall survival persisted for up to 7.5 years.^9^ Even in settings with homogeneity in disease stage, tumor biology, and prescribed treatments, our results show disparities in outcomes preferentially affecting those with less generous insurance types at trial registration.

Insurance may be a proxy for multiple domains of SDH and their effects on treatment completion or survival. For example, comorbidities such as obesity, hypertension, and cardiovascular disease are more prevalent in neighborhoods with healthy food deserts, a poorly structured built environment and diminished access to healthcare.^23^ Moreover, for racial and ethnic minorities, higher rates of comorbidities have been influenced by longstanding systematic discrimination and marginalization by governmental policies such as segregation and redlining which has adversely affected access to care.^24^ We hypothesize that insurance most likely serves as a proxy for the interaction between structural (governmental, economic and social policy etc.) and intermediary social determinants of health (i.e. working condition, financial hardship, transportation, social network, overall living conditions etc.).^1^, ^25^, ^26^ Nevertheless, we acknowledge comorbidities such as diabetes and cardiovascular disease, independent of SDH, contribute to drug toxicity which affects chemotherapy completion and survival.

We found no association between nSES and overall mortality, at odds with several prior studies on SES and mortality among breast cancer patients.^4^, ^27^, ^28^, ^29^ A meta‐analysis by Akinyemiju et al. suggests inconsistent nSES influence on mortality among breast cancer patients may be due to heterogeneity in indices used.^27^ A potential explanation for our results could be the use of the AHRQ SES index. The AHRQ SES index has been validated for use among Medicare patients^13^ and may not adequately capture the effects of nSES among nonelderly trial participants. Further, we may not have adequate power to detect nSES effects as our participants, similar to other trials, were younger, of a higher socioeconomic status and more educated than nonclinical trial counterparts.^7^ To better define the association of nSES with treatment completion or with mortality, the creation of an index capturing nSES of non‐Medicare populations or utilization of the same set of indices across multiple studies may be warranted.

The relationship between self‐pay and chemotherapy completion or mortality should be interpreted with caution. The self‐pay cohort in this study was very small and, therefore, may not be adequately powered to detect the difference between those with self‐pay versus private insurance.

The strengths of our study include the assessment of at least two domains of SDH on care delivery (i.e. treatment completion) and clinical outcomes (i.e. survival). We evaluated the effects of SDH in a clinical trial setting where participant and treatment homogeneity were expected to reduce the effects of SDH. Our limitations include the need for combining Medicaid and Medicare into one group. Medicaid and Medicare insure different sociodemographic populations. To address this issue, the multivariate analysis was adjusted for age, race, and nSES, which typically account for the main differences between Medicaid and Medicare patients. Moreover, due to strict clinical trial enrollment criteria, comorbidities would on average be no different across all insurance groups. Subset analyses of government insurance types in our data showed no differential outcome for Medicaid versus Medicare or for Medicaid plus Medicare versus other types of government insurance, again noting the numbers of patients with these insurance types were relatively small.

The Medicaid population in this study was enrolled prior to the January 2014 Medicaid Expansion under the Affordable Care Act (ACA) and, unlike the post‐ACA Medicaid population, are less healthy and have higher poverty levels.^30^, ^31^ It may be difficult to extrapolate these results to all government insured breast cancer patients.

## 5 CONCLUSION

For breast cancer patients enrolled in E1199 and E5103, government insurance (i.e. Medicare and Medicaid collectively) at trial registration was associated with decreased trial chemotherapy completion and increased overall mortality. Results from this study show that social determinants of health continue to influence outcomes even with strict clinical trial enrollment criteria for patients and similar treatment. Collection of a broader set of social determinants of health variables such as transportation, health literacy, employment status, and social networks is warranted to better define the impact on clinical trial participants and their outcomes.

## AUTHORS CONTRIBUTIONS

Conception or design of the work: Samilia Obeng‐Gyasi, Ruth Carlos, and Sheetal Kircher; Data collection: NA; Data analysis and interpretation: Anne O’Neill, Fengmin Zhao, Lava Timsina, Samilia Obeng‐Gyasi, Ruth Carlos, and Sheetal Kircher; Drafting the article: Anne O’Neill, Fengmin Zhao, Lava Timsina, Samilia Obeng‐Gyasi, Ruth Carlos, and Sheetal Kircher; Critical revision of the article: All listed authors; Final approval of the version to be published: All listed authors.

## Data Availability

The data that support the findings of this study are available from ECOG‐ACRIN. Restrictions apply to the availability of these data, which were used under license for this study. Data are available ECOG‐ACRIN with the permission of ECOG‐ACRIN.
